# Interdisziplinäre Chirurgie der zentralen Schädelbasis – aktueller Stand

**DOI:** 10.1007/s00106-021-01022-3

**Published:** 2021-04-06

**Authors:** J. Schipper, A. Albrecht, T. Klenzner, M. Wagenmann, K. Schaumann, D. Hänggi, J. F. Cornelius

**Affiliations:** 1 Universitätsklinik für Hals‑, Nasen- und Ohrenheilkunde und Poliklinik, Heinrich-Heine-Universität Düsseldorf, Moorenstraße 5, 40255 Düsseldorf, Deutschland; 2 Universitätsklinik für Neurochirurgie, Heinrich-Heine-Universität Düsseldorf, Düsseldorf, Deutschland

**Keywords:** Multi-Port-Zugang, Pathologien der zentralen Schädelbasis, Aktive Instumente, Minimal invasive Chirurgie, Mikrochirurgische Visualisierungsverfahren, Multiport access routes, Pathologies of the central skull base, Powered instruments, Minimally invasive surgery, Microsurgical visualization procedures

## Abstract

**Hintergrund:**

Die Schädelbasis stellt eine chirurgisch hochkomplexe Einheit dar und ist häufig nur über kombinierte Zugangswege erreichbar. Neu entwickelte Operationstechniken mit Verwendung von mikrochirurgischen Visualisierungsverfahren und aktiven Instrumenten („powered instruments“) sowie „Multi-Port-Zugänge“ ermöglichen neue, weniger traumatische Operationskorridore. Hierfür ist eine enge interdisziplinäre Zusammenarbeit zwischen dem Chirurgen aus dem Fachgebiet der Hals-Nasen-Ohren-Heilkunde sowie dem Neurochirurgen notwendig. Die aktuell etablierten Zugangsverfahren zur zentralen Schädelbasis werden aufgrund eigener klinischer Erfahrungen und unter Berücksichtigung der Entität systematisiert und in Bezug auf die aktuelle Studienlage erörtert.

**Material und Methode:**

Es erfolgte eine retrospektive, qualitative und deskriptive Auswertung der Operationsberichte einzelner Patienten, die in der Zeit zwischen 2006 und 2019 mit Pathologien an der zentralen Schädelbasis chirurgisch gemeinsam von der Neurochirurgie und der Hals-Nasen-Ohren-Heilkunde/Kopf- und Halschirurgie behandelt wurden.

**Ergebnisse:**

Die chirurgischen Zugangswege zur zentralen Schädelbasis ließen sich nachfolgend kategorisieren, teilweise auch in Kombination derselben, als sog. Multi-Port-Zugänge: transnasal-transsphenoidal, subfrontal, subtemporal, transzygomatisch, transpterygonal, transpetrös, translabyrinthär und subokzipital. Maßgebend für die Wahl des Zugangswegs waren die Lokalisation und Art der Pathologie, sowie der mögliche Anspruch auf Funktionserhalt und Komplettentfernung.

**Schlussfolgerung:**

Aufgrund der Komplexität der Strukturen der zentralen Schädelbasis, der unterschiedlichsten Tumorentitäten und der benötigten Fachkompetenz unterschiedlicher Facharztdisziplinen bleibt die Chirurgie der zentralen Schädelbasis eine Herausforderung, der man sich nur an speziellen, nach den Kriterien der Gesellschaft für Schädelbasischirurgie e. V. zertifizierten Kompetenzzentren stellen sollte.

## Abstract (en)

**Background:**

The skull base is a surgically complex unit and is often only accessible via combined access routes. Newly developed surgical techniques using microsurgical visualization procedures and active instruments (“powered instruments”) as well as multiport accesses enable new, less traumatic surgical corridors. This requires close interdisciplinary cooperation between ENT and neurosurgeons. Currently established access routes to the central skull base are systematized based on the authors′ own clinical experience, and discussed in relation to the entity and the current study situation.

**Materials and methods:**

A retrospective, qualitative, and descriptive evaluation of the surgical reports of patients with pathologies of the central skull base who were jointly treated by neurosurgery and otorhinolaryngologic/head and neck surgery between 2006 and 2019 was performed.

**Results:**

The surgical access routes to the central skull base can be categorized as so-called multiport access routes, partly also in combination, as follows: transnasal-transsphenoidal, subfrontal, subtemporal, transzygomatic, transpterygonal, transpetrous, translabyrinthine, and suboccipital. The choice of access route was based on the location and type of pathology, its inflammatory or space-occupying (benign or malignant tumor) nature, and the possibilities of functional preservation and complete removal.

**Conclusion:**

Due to the complexity of central skull base structures, the different tumor entities, and the required expertise of different medical specialties, surgery of the central skull base remains a challenge and should only be performed at special competence centers certified according to the criteria of the German Society of Skull Base Surgery.

Die zentrale Schädelbasis definiert sich chirurgisch-anatomisch aus der Felsenbeinspitze, dem Clivus, dem medialen und lateralen Keilbeinflügel mit dem Nasopharynx sowie aus dem kraniozervikalen Übergang mit dem dentoatlantovertebralen Gelenkkomplex. Der Zugang zu dieser chirurgisch komplexen Einheit gelingt selten über einen einzelnen Operationsweg. Häufig ist er nur kombiniert über die Fronto- oder Laterobasis sowie zusätzlich über die natürlichen Körperhöhleneingänge von Nase und Mund erreichbar [2, 28]. Durch diese Kombination und durch gängige Visualisierungsverfahren konnten auch 4‑Hand-Techniken etabliert werden, die Op.-Sicherheit, -Durchführbarkeit erheblich vereinfacht und verbessert haben.

Mit der Weiterentwicklung mikrochirurgischer Visualisierungsverfahren als Alternative zum Operationsmikroskop, wie die Einführung kameragestützter Winkeloptiken und dem Einsatz aktiver Instrumente („powered instruments“) wie dem CUSA („cavitron ultrasonic surgical aspirator“) oder Shaver, haben sich in dem letzten Jahrzehnt neue, weniger traumatische Zugangswege ergeben [1]. Hierdurch ist es zum einen möglich, bislang als nicht sinnvoll operable Pathologien chirurgisch zu sanieren, und zum anderen, mögliche Kollateralschäden deutlich zu begrenzen.

Für die Entwicklung neuer chirurgischer Zugangswege zur zentralen Schädelbasis haben sich umfangreiche vorangegangene anatomisch-chirurgische Kadaverstudien sehr bewährt [2, 29, 31]. Darüber hinaus führen solche Kadaverstudien auch zu einer Verbesserung der Lernkurve, bevor es in den Operationssaal geht. Allerdings ergeben sich bei der klinischen Umsetzung solcher neu zu etablierender Zugangswege, auch bei noch so guter Vorbereitung und Präparation, nicht vorhersehbare Probleme. Insbesondere venöse Sickerblutungen aus den unterschiedlichsten Weichteilstrukturen mit Verlegung der endoskopischen oder mikroskopischen Sicht stellen dabei gelegentlich eine Herausforderung dar. Dies kann dann dazu führen, dass unter Umständen neue Operationskorridore sich in der klinischen Praxis entgegen den Erfahrungen aus den Kadaverstudien doch nicht bewähren und man auf bisher etablierte Operationsverfahren wieder zurückgreift. Auch solche Studien- und Erfahrungsergebnisse, auch wenn sie unter Umständen Irrwege darstellen, sind für die Weiterentwicklung der Schädelbasischirurgie von erheblicher Bedeutung.

Die Entwicklung sog. neuer einzeitiger oder mehrzeitiger „Multi-Port-Zugänge“ im Vergleich zu den bisherigen konventionellen „Single-Port-Zugängen“ ermöglichen durch kombinierten Einsatz von Mikroskop und Endoskop mit Videoketten zur Visualisierung des Operationssitus neue perspektivische Aufsichten sowohl auf die Zielpathologie als auch auf die den chirurgischen Manipulationskorridor verlegenden vitalen Strukturen wie Hirnnerven oder die A. carotis interna. So kann man etwa neben dem Manipulationskorridor durch Präparation eines oder mehrerer zusätzlicher minimal-invasiver Gewebstunnel mit Einführen einer 30- oder 45°-Optik die Zielpathologie oder andere bedeutsame Strukturen erstmalig zusätzlich zum jeweiligen Manipulationskorridor auch von der Seite oder von hinten betrachten [28]. Dieser perspektivische „Kubismus“ der jeweiligen Zielstruktur ermöglicht eine noch sicherere chirurgische Exploration im Bereich der zentralen Schädelbasis. Der obligatorische Einsatz der Navigation dient dabei nur der Plausibilisierung des jeweiligen Operationsschritts. Geführt wird der Operateur dabei ausschließlich durch die Visualisierung des jeweiligen Operationssitus und der so sichtbar werdenden Anatomie mit ihren Leitstrukturen. Die Hersteller von Mikroskopen oder anderer Visualisierungssysteme haben sich bereits auf diese neuen Operationstechniken eingestellt und bieten beispielsweise Mikroskope in Kombination mit flexiblen Endoskopen oder sog. stationären Exoskopen in Kombination mit Endoskopen in 4K-Technologie an. Sämtliche verfügbaren perspektivischen Bilddaten inkl. Messdaten vom Neuromonitoring oder von der Navigation werden dann in einer für den Operateur optimalen ergonomischen Position auf einen einzigen Masterbildschirm projiziert [33].

Ein nächster Schritt in die Zukunft wird bei mehrzeitigen Multi-Port-Zugängen die „geodetected navigated visualisation“ sein, das bedeutet, dass korrespondierend zu dem navigierten Zielpunkt das entsprechende intraoperative Bild oder die Videosequenz aus der Voroperation neben dem aktuellen intraoperativen Situs eingeblendet wird. Durch den Vergleich mit den historischen Bilddaten aus einer möglichen anderen Perspektive erhält der Operateur wertvolle Informationen für das weitere mehrzeitige Vorgehen. Allerdings verlangt eine solche Technologie erhebliche Speicherkapazitäten sowie sehr schnelle Grafikkarten, die derzeit noch nicht flächendeckend vorhanden sind. Durch eine detaillierte Auflösung des „fibertracking“ wird es in der Zukunft präoperativ wertvolle Hinweise über den Verlauf der neuronalen Bahnen für die Op.-Planung geben. Der naturgemäß deutlich verlängerte Zugangsweg zur zentralen Schädelbasis, ob nun ausgehend von der Fronto- oder Laterobasis oder vom Splanchnokranium kommend, engt den chirurgischen Manipulationskorridor deutlich ein, wenn man nicht andere den Zugangsweg kreuzende Vitalstrukturen unnötig invalidisieren will. Die Präparation an der Zielpathologie ist damit durch den Eingangsdurchmesser des Manipulationskorridors und der sich daraus ergebenden verkürzten Hebelwege eingeschränkt. „Powered instruments“ wie der CUSA („cavitron ultrasonic surgical aspirator“) oder Shaver sind für den notwendigen Gewebsabraum der Zielpathologie sehr hilfreich und schaffen dadurch gleichzeitig Platz für die weitere chirurgische Manipulation und Visualisierung [1]. Sie eignen sich jedoch je nach Konsistenz und Vaskularisierungsgrad nicht für jede Zielpathologie. Bei stark vaskularisierten Zielpathologien im Bereich der zentralen Schädelbasis reduziert eine präoperative endovaskuläre Embolisation das „red-out“ und ermöglicht eine operative Entfernung.

Allen neu entwickelten Operationsverfahren gemeinsam ist die Notwendigkeit einer engen interdisziplinären Zusammenarbeit zwischen dem Chirurgen aus dem Fachgebiet der Hals-Nasen-Ohren-Heilkunde sowie dem Neurochirurgen, da die Pathologien im Bereich der zentralen Schädelbasis nicht haltmachen vor den chirurgischen Grenzen der deutschen Facharztweiterbildung. Die Art und Ausdehnung des notwendigen Operationskorridors werden dabei im Wesentlichen durch die präoperativ radiologisch vermutete Entität der Pathologie sowie durch deren topographische Ausdehnung in der zentralen Schädelbasis bestimmt. Wir haben daher aufgrund unserer eigenen klinisch-chirurgischen Erfahrungen der letzten 14 Jahre die jetzt etablierten Zugangsverfahren zur zentralen Schädelbasis unter Berücksichtigung der Entität systematisiert und erörtert auf Grundlage der aktuell publizierten Studienlage.

## Patienten und Methoden

Zur Systematisierung und Würdigung der verschiedenen zu erörternden operativen Zugangswege zur zentralen Schädelbasis wurden retrospektiv, ausschließlich qualitativ und deskriptiv die Operationsberichte einzelner Patienten ausgewertet, die laut Beschluss der hiesigen interdisziplinären Schädelbasiskonferenz gemeinsam mit der Neurochirurgie und der Hals-Nasen-Ohren-Heilkunde/Kopf- und Halschirurgie in der Zeit zwischen 2006 und 2019 mit Pathologien an der zentralen Schädelbasis chirurgisch behandelt wurden. Für eine Systematisierung wurden die Kriterien chirurgischer Zugangsweg, komplette oder inkomplette Sanierung der Pathologie, Art des multimodalen Therapiekonzepts (Chirurgie als alleinige, neoadjuvante oder adjuvante Therapiemaßnahme) sowie Erst- oder Rezidivtherapie erfasst. Chirurgisches Ziel jeder malignen Pathologie im Bereich der zentralen Schädelbasis war die komplette Entfernung oder aber auch die Diagnosesicherung bei systemischen malignen Erkrankungen. Lediglich bei den benignen Pathologien einschließlich entzündlicher Prozesse war zum Funktionserhalt endokranieller neurovaskulärer Strukturen eine inkomplette bzw. subtotale Resektion unter Umständen angestrebt.

## Darstellung und Diskussion der Ergebnisse

**Figure 1:**
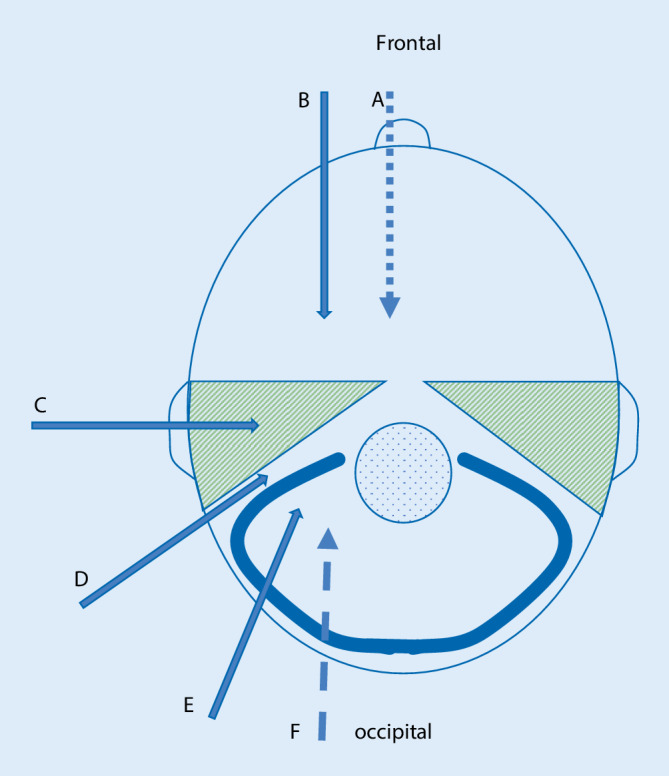

Die chirurgischen Zugangswege zur zentralen Schädelbasis ließen sich nachfolgend kategorisieren, teilweise auch in Kombination derselben, als Multi-Port-Zugänge: transnasal-transsphenoidal, subfrontal, subtemporal, transzygomatisch, transpterygonal, transpetrös, translabyrinthär und subokzipital (Abb. 1). Maßgebend für die Wahl des Zugangswegs waren die Lokalisation und Art der Pathologie, ob entzündlich oder raumfordernd (benigner oder maligner Tumor) sowie der mögliche Anspruch auf Funktionserhalt und Komplettentfernung (Tab. 1). Maligne Pathologien der zentralen Schädelbasis wachsen im Gegensatz zu den benignen Pathologien lokal destruierend und können je nach Entität auch metastasieren. Häufigste maligne Pathologien im Bereich der zentralen Schädelbasis sind Karzinome und Sarkome. Bei den Karzinomen finden sich in erster Linie Plattenepithelkarzinome und Adenokarzinome, seltener adenoidzystische Karzinome oder Metastasen, sowie nachfolgend Meningeome (WHO-Grad III, World Health Organization). Bei den Sarkomen sind an erster Stelle die Chondrosarkome zu nennen.

**Table 1:** 

**Entität**	Chirurgischer Zugangsweg und Visualisierungssystem (endoskopisch: E, mikroskopisch: M)	Therapiestrategie	Adjuvante Therapie
**Histopathologische Entität**			
**Hypophysenadenome**	Transnasal-transsphenoidal *E/M*	Komplette Resektion ^b^	Bei Residuum/Rezidiv: (stereotaktische) Strahlentherapie (SRS/SRT)
**Frontobasale Meningeome**	Transkraniell (z. B. „Trans-Eyebrow-Zugang“, Minikraniotomien) *M*, ggf. *E* assistiert	Komplette Resektion ^b^	Bei Residuum/Rezidiv od. höherem WHO-Grad: (stereotaktische) Strahlentherapie (SRS/SRT)
	Transnasal-transplanum *E*		
**Chordome**			
Median gelegene, kleine bis mittelgroße Tumoren	Transnasal, transclival, ggf. ergänzt durch transoral *E*	Radikalchirurgische Resektion ^a^	Hochenergetische Partikelbestrahlung
Große Tumoren	Multi-Port-Zugang: transnasal, transsphenoidal, transfazial, transclival *E*		
	Transzervikal, transkondylär (z. B. „Far-Lateral-Zugang“) *M* ggf. mehrzeitiges Vorgehen		
	Häufig mit kraniozervikaler Stabilisierung		
**Extrakranielle Neurinome/Paragangliome**	Transzervikal *M*	Komplette Resektion ^b^	Bei Residuum/Rezidiv: (stereotaktische) Strahlentherapie (SRS/SRT)
**Entzündliche Veränderungen**	Transmastoidal *M*, ggf. ergänzt durch transnasal *E*	Abstrichentnahme, chirurgische Abtragung von Knochensequestern, Drainage	Langzeitantibiose, ggf. HBO-Therapie
**Topographische Entität**			
**Pathologien der Felsenbeinspitze**			
Dermoide	Subtemporal *M*	Komplette Resektion ^b^	–
Genuine Cholesteatome			–
Chondrosarkome		Radikalchirurgische Resektion ^a^	Ggf. hochenergetische Partikelbestrahlung
Metastasen			–
Cholesteringranulome	Ggf. transnasal-transsphenoidal *E*	Ggf. Drainage ausreichend	–
**Pathologien im Bereich des Sinus cavernosus**			
Trigeminusneurinome	Subtemporal (extra- oder intradural) *M*	Komplette Resektion ^b^	Bei Residuum/Rezidiv: (stereotaktische) Strahlentherapie (SRS/SRT)
Chondrosarkome	Subtemporal (extra- oder intradural), ggf. transzygomatisch *M*	Radikalchirurgische Resektion ^a^	Ggf. hochenergetische Partikelbestrahlung
Riesenzellgranulome		Komplette Resektion ^b^	–
Osteoblastome			–
Fibröse Dysplasie			Ggf. Bisphoshonate
**Tumoren medial der Gl. parotis**			
Pleomorphe Adenome	Transmaxillär* M/E*, transpterygonal, ggf.* E*	Radikalchirurgische Resektion ^a^	–
Adenokarzinome			–
Plattenepithelkarzinome			Ggf. Radio‑/Chemotherapie

*SRS* stereotaktische Radiochirurgie, *SRT* fraktionierte stereotaktische Strahlentherapie

^a^ Hiermit ist meistens keine R0-Resektion im onkochirurgischen Sinne gemeint, da durch Sicherheitsabstände im Bereich der Schädelbasis häufig ein zu hoher Grad an Invalidisierung erreicht würde. Es muss häufig ein Kompromiss zwischen notwendiger Radikalität und Funktionserhalt getroffen werden

^b^ Zum Funktionserhalt ist u. U. auch eine inkomplette Resektion möglich

Transnasale bzw. transnasal-transsphenoidale Zugänge wurden bei benignen und malignen Raumforderungen eingesetzt, welche lokal auf den Clivus sowie den kraniozervikalen Übergang begrenzt waren und jeweils medial der A. carotis interna (ACI) lagen. Typische benigne Tumoren sind *Hypophysenadenome, frontobasale Meningeome *sowie* Kraniopharyngeome*. Zu den malignen Tumoren der Region zählen u. a. *lokal aggressive Chordome,* *Ästhesioneuroblastome, Plattenepithel- *und *Adenokarzinome*. Mit Infiltration der Dura, aber ohne wesentliche Ausdehnung nach intrazerebral, werden sie üblicherweise transnasal reseziert. Die eingeschränkte Möglichkeit der chirurgischen Manipulation und Kontrolle, insbesondere bei Blutungen lateral der ACI, wurden bereits ausführlich durch die Publikationen von Kassam u. a. erörtert, [9–11] sodass man bei Raumforderungen mit Ausdehnung über die ACI nach lateral hinaus im Allgemeinen einen transkraniellen Zugangsweg favorisieren würde. Seltene Ausnahmen stellen im Einzelfall nur benigne Tumoren dar.

*Hypophysenadenome* können als der Prototyp für den transnasalen endoskopischen Zugangsweg angesehen werden [21, 22]. Dieser kann mono- oder bilateral erfolgen, was v. a. von Größe und Konsistenz des Tumors abhängt. Der Zugang muss so groß wie nötig sein, um den Tumor sicher und komplett entfernen zu können. Andererseits ist aber für den Erhalt einer guten Lebensqualität ein möglichst geringes Zugangstrauma in der Nase wichtig, d. h. Schonung und Erhalt von Nasenschleimhaut, -septum und -muscheln. Die knöcherne bzw. durale Rekonstruktion der Schädelbasis erfolgt in Abhängigkeit von der Defektgröße und dem erwarteten Risiko einer Durafistel [19]. Bei kleineren Defekten mit niedrigem Liquorfluss sind kleinere Abdichtungsmaßnahmen meist ausreichend. Bei größeren Defekten und hohem Liquorfluss, z. B. nach Eröffnung des III. Ventrikels und des Diaphragma sellae, kann durch einen gestielten, vaskularisierten mukoseptalen Lappen n. Hadad im Allgemeinen ein wasserdichter Verschluss gut erreicht werden [3].

Frontobasale *Meningeome*, uni- oder bilateral der Riechspalte, wurden nach Einführung und stetiger Verbesserung der endoskopischen Techniken zeitweise bevorzugt transnasal operiert. Aufgrund des hierbei erhöhten Risikos einer Hyp- oder gar Anosmie hat es im Laufe der Zeit dann wieder eine Rückkehr zu den transkraniellen Zugängen gegeben [22]. Die transkraniellen Zugänge haben sich allerdings im Vergleich zu den Anfängen der Schädelbasischirurgie zunehmend zu weniger invasiven Varianten entwickelt, „Keyhole-Konzept“ [4, 20, 30]. Kleine Hautschnitte, z. B. ein „Trans-Eyebrow-Zugang“ sowie Minikraniotomien führten zu einer deutlichen Verringerung des Zugangstraumas mit einfacheren und schnelleren postoperativen Heilungsverläufen bei ästhetisch sehr guten Ergebnissen [5, 6]. Die durch Miniaturisierung der Zugänge reduzierte Sicht kann durch ein Endoskop gut kompensiert werden, sog. endoskopisch-assistierte Mikrochirurgie.

Bei den* Chordomen *handelt es sich um maligne Knochentumoren mit notochordaler Differenzierung, die sich fast ausschließlich entlang des Achsenskeletts entwickeln. Sie haben ihre Ursprungsmatrix in der phylogenetisch sich bildenden Neuralleiste. Die Neuralleiste wiederum befindet sich in der Medianebene am kraniozervikalen Übergang. Daher befinden sich Chordome im Bereich der Schädelbasis typischerweise im Clivus, von wo aus sie dann lokal destruierend in alle Richtungen wachsen [13]. Grundsätzlich sollte aufgrund der sehr hohen Rezidivneigung von Chordomen immer eine radikale Tumorentfernung angestrebt werden, auch deswegen, da die Chordome relativ schlecht durch Strahlen- und Chemotherapie allein therapierbar sind [18].

Häufig ist die Stabilität des kraniozervikalen Übergangs bereits durch die Tumordestruktion beeinträchtigt, manchmal wird diese aber auch durch die notwendige radikale Resektion induziert und muss in jedem Fall durch eine kraniozervikale Stabilisierung behandelt werden.

**Figure 2:**
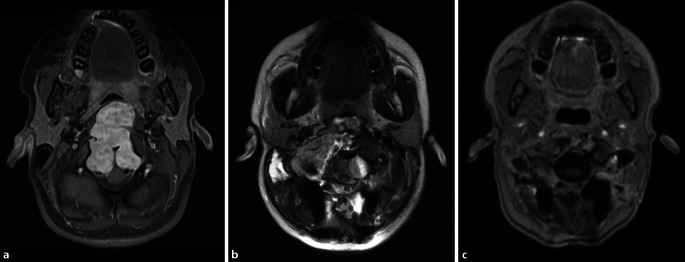

Für median gelegene, kleine bis mittelgroße Chordome ist meist ein transnasal-transclivaler, ggf. ergänzt durch einen transoralen Zugangsweg geeignet. Große Chordome (Abb. 2 und 3) müssen unter Umständen über einen Multi-Port-Zugang reseziert werden, ggf. sogar mehrzeitig, beispielsweise kombiniert transnasal, transzervikal und/oder transkondylär. Die adjuvante Therapie der ersten Wahl ist derzeit die postoperative hochenergetische Partikelbestrahlung durch Protonen oder Karbonionen [17]. Da die Radiatio durch etwaiges Osteosynthesematerial beeinflusst werden kann, ist für Chordome eine enge interdisziplinäre Zusammenarbeit und eine sorgfältige multimodale Therapieplanung von besonderer Bedeutung [24]. Die Überlebensprognose der Patienten ist bei einer radikalen Therapie bei Erstmanifestation am besten, weshalb die Behandlung der extrem seltenen Chordome Schwerpunktzentren vorbehalten bleiben sollte [14–16].

**Figure 3:**
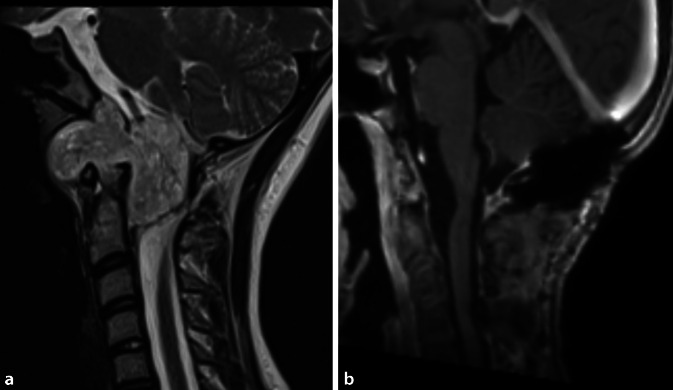

Pathologien im Bereich der Felsenbeinspitze lassen sich gut von lateral kommend über einen subtemporalen Zugangsweg resezieren. Da bei diesem Zugangsweg nicht der Meatus acusticus internus das Zielgebiet ist, sondern die Felsenbeinspitze, wird die Kraniotomie temporobasal etwas weiter frontal durchgeführt und der weitere Zugang dann extradural unterhalb des Temporallappens [27]. Dieser sollte beim Lagern durch eine leichte Kopfflexion bodenwärts dazu gebracht werden, sich der Schwerkraft folgend vom Boden der mittleren Schädelgrube abzuheben. Dies kann durch eine zusätzliche Hirnrelaxation mit Lumbaldrainage und vorheriger Mannitgabe unterstützt werden. Das Einsetzen eines Spatels sollte möglichst vermieden werden, um den Temporallappen nicht zu schädigen. Weiterhin muss besonders auf das horizontale bzw. petrosale Segment der A. carotis interna Rücksicht genommen werden, welche im Felsenbein nur durch einen dünnen Knochen bzw. bisweilen gar nicht knöchern gedeckt verläuft. Typische Pathologien sind gutartige Tumoren wie* Dermoide, genuine Cholesteatome, Cholesteringranulome, *aber auch maligne Raumforderungen wie* Chondrosarkome oder Metastasen.* Bei den *Cholesteringranulomen* ist zu bedenken, dass eine sichere vollständige Resektion nicht immer möglich ist und an eine ausreichende Drainage für das nachlaufende Zellsekret aus den verbleibenden Granulomzellen entweder nach transnasal oder transtympanal gedacht werden muss. In diesen Fällen kann auch, je nach anatomischer Situation, eine transnasal-transsphenoidale Exploration der Felsenbeinspitze von Vorteil sein, ggf. auch als Multi-Port-Zugang [23, 25]. Bei der Wahl des transnasalen Zugangswegs ist v. a. die präoperativ computertomographisch zu bestimmende Größe der lateralen Wand des Sinus sphenoidalis sowie dessen topographische Lage zum Recessus caroticoopticus entscheidend, inwieweit ein solcher Zugangsweg überhaupt möglich ist.

**Figure 4:**
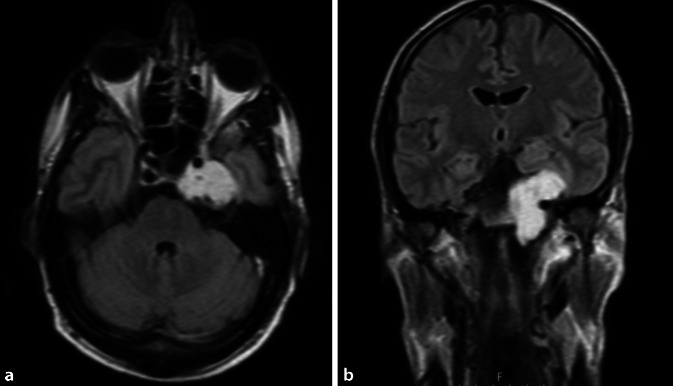

Der subtemporale Zugangsweg kann durch einen transzygomatischen Zugang erweitert werden. Diese Kombination ermöglicht es dann gut, zentral gelegenen Pathologien im Bereich des Sinus cavernosus mit Extension in die Fossa pterygopalatina zu erreichen (Glascock-Dreieck). In erster Linie erfolgt auch dieser Zugang extradural und kann aber im Zielbereich ggf. durch ein intradurales Vorgehen ergänzt werden. Neben den typischen Trigeminusneurinomen und Chondrosarkomen gibt es andere seltenere Pathologien in diesem Bereich, wie z. B. *Riesenzellgranulome, Osteoblastome *oder eine* fibröse Dysplasie*. Die Schlüsselprozedur bei diesem Zugangsweg ist die temporäre Rotation des vorderen Temporalmuskelanteils nach frontokaudal unter Schonung des Ramus frontalis nervi facialis sowie die osteoplastische Transposition des Os zygomaticum [26]. Damit erhält man einen optimalen Überblick auf die Seitenwand des Sinus cavernosus sowie auf die kranialen Strukturen der Fossa pterygopalatina, ohne dass man dadurch die Substanz und Funktion der Pterygoidmuskulatur verletzt. Dieses kann bleibende Funktionsstörungen der Kaumuskulatur nach sich ziehen (Abb. 4 und 5). Ebenso ist hierdurch auch keine Sichteinschränkung durch den Processus coronoideus gegeben. Tiefer gelegene Pathologien im Bereich der mittleren oder unteren Etage der Fossa pterygopalatina lassen sich dagegen besser transnasal, transpterygonal bzw. transmaxillär endoskopisch angehen. Lediglich nachteilig dabei ist unter Umständen die Traumatisierung und anschließende Vernarbung der Pterygoidmuskulatur mit nachfolgenden Einschränkungen in der Kiefergelenkbewegung. Zudem kommt es durch diffuse Sickerblutungen im Bereich des venösen Plexus pterygoideus häufig zur endoskopischen Sichteinschränkung, die diesen Zugangsweg dann zu einer chirurgischen Geduldsprobe werden lassen. Die A. maxillaris wird in der Regel geclippt. Der radiologisch sichtbare Clip ist dann später im Follow-up eine wichtige Landmarke zur topographischen Orientierung in dem chirurgisch veränderten Bilddatensatz. Der N. vidianus ist intraoperativ häufig die bessere Nervenleitstruktur als die operative Navigation zur Identifizierung der daruntergelegenen A. carotis interna [11, 12].

**Figure 5:**
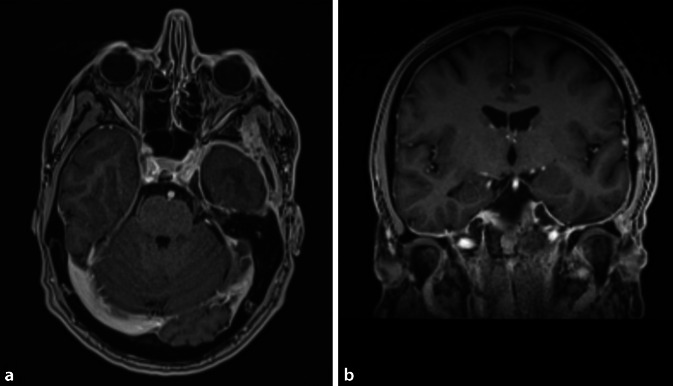

Anders als bei raumfordernden Tumorentitäten gestaltet sich das Therapieregime bei entzündlichen Veränderungen im Bereich der zentralen Schädelbasis. In der Regel handelt es sich dabei um eine Osteomyelitis oder um eine Ostitis, die radiologisch je nach Entzündungsstadium nicht immer sofort erkennbar sind. Häufig sind dabei klinische Prodromalzeichen wegweisend wie etwa das bekannte „Gradenigo-Syndrom“ oder andere solitäre Hirnnervenausfälle in Kombination mit Kopf- und Gesichtsschmerzen. Ausgangspunkt kann eine Sinusitis oder eine Otitis externa necroticans sein, möglicherweise begünstigt durch eine immunsuppressive Abwehrlage oder bei Diabetes mellitus. Durch die Unspezifität der klinischen Symptomatik sind Patienten oft bereits mit Breitbandantibiotika frustran vorbehandelt, was eine spätere mikrobiologische Charakterisierung zusätzlich erschwert. Darüber hinaus muss man erst einmal durch einen operativen Zugangsweg zur zentralen Schädelbasis die Möglichkeit schaffen, entzündlich abgekapseltes Gewebe zu entfernen und den Bereich sozusagen zu „belüften“. Dies gelingt häufig nur durch ein kombiniert chirurgisches Vorgehen – transmastoidal im Sinne einer Petrosektomie und transnasal über die Keilbeinhöhle und den Nasopharynx. Nur so kann ein repräsentativer Abstrich am Zielort auch von abgekapselten Prozessen aus dem Knochenmaterial gewonnen werden. Ursächlich sind hier häufig Anaerobier oder auch *Pseudomonas aeruginosa* im Rahmen einer Otitis externa maligna. Neben der notwendigen chirurgischen Abtragung von Knochensequestern und Drainierung zur äußeren Umgebung bei bakteriellen Infekten im Bereich der zentralen Schädelbasis kann v. a. bei Anaerobiern der Einsatz der hyperbaren Sauerstofftherapie den Genesungsprozess deutlich beschleunigen [7, 8]. In der Regel verlaufen solche Erkrankungen schleichend progredient sowie langwierig und bedürfen daher einer äußerst langen begleitenden Antibiotikatherapie, teilweise über mehrere Monate bis Jahre, einschließlich radiologischer Kontrollen. Zusätzlich fortschreitende Infektionen mit Pilzen im Bereich der zentralen Schädelbasis stellen eine besondere Herausforderung dar und müssen radikalchirurgisch saniert werden, da in solchen Fällen die Mortalität deutlich ansteigt [7].

Medial der Gl. parotis gelegene Tumoren, lateral oder medial eines der beiden Pterygoidflügel wie *pleomorphe Adenome* aber auch *Adeno- oder Plattenepithelkarzinome* lassen sich über einen transmaxillär-transpterygonalen Zugangsweg endoskopisch-chirurgisch erfassen. Bei den malignen Tumoren ist im Rahmen multimodaler Therapiekonzepte immer eine radikalchirurgische Sanierung anzustreben. Eine „Piecemeal-Technik“ schließt dies aber nicht aus. Chirurgisch-endoskopische Leitstruktur ist dabei der N. infraorbitalis der mit endoskopischer Entfernung des pterygomaxillären Pfeilers in die Fossa pterygopalatina verfolgt wird bis hin zum Ganglion pterygopalatinum. Der anterior des Ganglion pterygopalatinum gelegene Plexus venosus pterygopalatinum verlegt häufig durch venöse Sickerblutungen die endoskopische Sicht und kann den Fortgang der Operation erheblich verzögern. Größere maligne Tumoren im Bereich der Fossa pterygopalatina sollten je nach multimodalem Tumorkonzept ggf. auch extranasal über eine temporäre osteoplastische Maxillektomie mit Exploration der Fossa pterygopalatina radikalchirurgisch entfernt werden. Eine interdisziplinäre Einbindung der Neurochirurgen für medial der Gl. parotis gelegene Pathologien ist immer dann von Bedeutung, wenn eine Extension nach endokraniell in der präoperativen Bildgebung gesehen oder zumindest stark angenommen wird.

Extrakraniell gelegene *Neurinome* des N. hypoglossus oder des N. vagus oder *vagale Paragangliome*, aber auch seltenere *ektope Meningeome* vergleichbarer Lokalisation können auch alternativ über einen medial des M. digastricus zu präparierenden Zugangsweg angegangen werden. Dabei wird zur Sicherung der A. carotis interna die Fascia cervicalis profunda entlang der Gefäßscheide nach kranial verfolgt bis hin zur Schädelbasis medial des Processus stylomastoideus, um dann den Tumor von kranial nach kaudal zu mobilisieren und zu entfernen. Zur Schonung der kaudalen Hirnnerven sollte in diesem Bereich immer ein entsprechendes intraoperatives Neuromonitoring sichergestellt sein.

## Schlussfolgerung

Maligne Tumoren bedürfen aus onkologischer Sicht immer einer radikalchirurgischen Entfernung, benigne Tumoren können unter Umständen zum Funktionserhalt auch inkomplett reseziert werden. Da die zu behandelnden Pathologien im Bereich der zentralen Schädelbasis häufig eine unmittelbare Nachbarschaft zu vitalen Strukturen aufweisen, müssen zur Wahrung der Lebensqualität im Rahmen multimodaler Tumortherapiekonzepte auf Basis belastbarer Studien in Kombination mit nichtchirurgischen, neoadjuvanten oder adjuvanten Behandlungsverfahren entsprechende Kompromisse entwickelt werden. Diese müssen vor Beginn einer solchen Behandlung in einer durch die Deutsche Gesellschaft für Schädelbasischirurgie (DGSB) zertifizierten, interdisziplinären Schädelbasiskonferenz auf Grundlage der radiologisch-klinischen Diagnostik erörtert und festgelegt werden. Nicht zuletzt wegen möglicher späterer medikolegaler Haftungsansprüche sind diese Entscheidungen immer transparent und nachvollziehbar im Rahmen der notwendigen interdisziplinären Schädelbasiskonferenzen zu dokumentieren.

In der Regel sind die Entscheidungen der interdisziplinären Schädelbasiskonferenz sehr individuell. Damit ist die Möglichkeit einer Stratifizierung der verschiedenen Tumorentitäten der Schädelbasis für eine Studie statistisch erheblich eingeschränkt. Vor allem ist aufgrund individuell unterschiedlicher Tumorwachstumsmuster letztlich keine sinnvolle Kategorisierung möglich. Nicht zuletzt auch deswegen gibt es für die Malignome im Bereich der zentralen Schädelbasis keine TNM-Klassifikation der WHO. Grundlage jeder therapeutischen Entscheidung ist somit v. a., inwieweit die beteiligten interdisziplinären chirurgischen Fachabteilungen sich in der Lage sehen, die jeweilige Pathologie komplett oder inkomplett chirurgisch zu sanieren. Davon abhängig erfolgt dann die Anpassung einer neoadjuvanten oder adjuvanten Strahlen- oder kombinierten Strahlen‑/Chemotherapie bei den malignen Neoplasien [32]. Die chirurgische Radikalität muss darüber hinaus gegen den zu erwartenden, funktionell-chirurgischen Flurschaden abgewogen werden.

Aufgrund der Komplexität der Strukturen der zentralen Schädelbasis, der unterschiedlichsten Tumorentitäten und der benötigten Fachkompetenz unterschiedlicher Facharztdisziplinen bleibt die Chirurgie der zentralen Schädelbasis eine Herausforderung, der man sich nur an speziellen zertifizierten Kompetenzzentren stellen sollte.
